# Single Image Super-Resolution Based on Multi-Scale Competitive Convolutional Neural Network

**DOI:** 10.3390/s18030789

**Published:** 2018-03-06

**Authors:** Xiaofeng Du, Xiaobo Qu, Yifan He, Di Guo

**Affiliations:** 1 School of Computer and Information Engineering, Xiamen University of Technology, Xiamen 361024, China; xfdu@xmut.edu.cn (X.D.); y.he@xmut.edu.cn (Y.H.); 2 Department of Electronic Science, Xiamen University, Xiamen 361005, China; quxiaobo@xmu.edu.cn

**Keywords:** multi-scale, convolutional neural network, image super-resolution

## Abstract

Deep convolutional neural networks (CNNs) are successful in single-image super-resolution. Traditional CNNs are limited to exploit multi-scale contextual information for image reconstruction due to the fixed convolutional kernel in their building modules. To restore various scales of image details, we enhance the multi-scale inference capability of CNNs by introducing competition among multi-scale convolutional filters, and build up a shallow network under limited computational resources. The proposed network has the following two advantages: (1) the multi-scale convolutional kernel provides the multi-context for image super-resolution, and (2) the maximum competitive strategy adaptively chooses the optimal scale of information for image reconstruction. Our experimental results on image super-resolution show that the performance of the proposed network outperforms the state-of-the-art methods.

## 1. Introduction

As one of the classical yet challenging problems in image processing, the goal of single-image super-resolution (SISR) is to restore a high-resolution (HR) image from a low-resolution (LR) image input by inferring all the missing high-frequency details. Super-resolution is also a crucial step in many real-world applications, e.g., security and surveillance imaging, television display, satellite imaging and so on.

However, the image super-resolution problem is an inherently ill-posed problem because many HR images can be down-sampled to the same LR image. Such a problem is typically mitigated by constraining the solution space by strong prior information, which assumes that the neighborhood of a pixel provides reasonable information to restore high-frequency details that lost by down-sampling. For a detailed review of these methods see [1]. In general, the current approaches for super-resolution can be categorized into three classes: interpolation-, reconstruction-, and learning-based methods [2,3,4,5,6,7,8]. Recently, learning-based methods [9,10,11] achieved state-of-the-art performance. The above methods typically work at the level of small fixed-size image patches.

Recent years have witnessed significant advancement in speech and visual recognition with deep convolutional neural networks (CNNs) [12,13,14]. CNN consists of multiple convolutional layer. Benefiting from the large number and size of the convolutional kernels in each convolutional layer, CNN has strong learning capacity and can automatically learn hierarchies feature from training data [15]. In the task of image super-resolution, Dong et al. proposed a deep learning-based method named super-resolution CNN (SRCNN) [16,17]. Compared with previous learning-based approaches, SRCNN exploits more contextual information to restore lost image details and achieves leading performance. In general the effective size of image context for reconstruction is correlates with the receptive field size of CNN [18]. Specifically, the receptive field size of SRCNN depends on the convolutional kernel size in each layer and the depth of CNN. Kim et al. developed a very deep CNN [19], which enlarges the receptive field size by stacking more convolutional layers. Yamamoto et al. successfully introduced deep CNN-based super-resolution to agriculture [20]. However, both larger kernel size and deeper network bring more parameters and consume more computing resources. Moreover, once the kernel scale and the depth are fixed, CNN only provides single scale contextual information for image reconstruction, which is ignorant of the inherent multi-scale nature of real-world image.

Each image feature has its own optimal scale at which the image feature is the most pronounced and distinctive from its surroundings. Considering that HR image restoration may rely on both short- and long-range contextual information, an ideal CNN should adaptively determine the convolutional kernel with a large scale on smooth regions and a small scale on texture regions possessing abundant details. On one hand, convolutional layer with large scale kernel has the capability to learn complex features but has more parameters. On the other hand, small scale of convolutional kernel makes CNN more compact thus easy to learn, but has less ability to represent the image features.

A practice solution is to adopt multi-scale inference [21,22,23,24,25] into CNN, yielding two questions: How to introduce multi-scale convolution into CNN and how to choose an optimal scale of the convolutional kernel? In this paper, we introduce a new module to tackle these questions. The proposed module is composed of multi-scale convolutional filters joined by a competitive activation unit.

The contributions of this paper include:

- We introduce multi-scale convolutional kernel to traditional convolutional layers, which provides multi-range contextual information for image super-resolution;
- We adopt a competitive strategy to CNN, which not only adaptively choose the optimal scale for convolutional filters but also reduces the dimensionality of the intermediate outputs.

The remainder of this paper is organized as follows. The related works are reviewed in Section 2. In Section 3, the structure and training process of our multi-scale CNN are discussed in detail. Section 4 presents the experimental results on image super-resolution. Section 5 discuss the comparisons with other state-of-the-art methods and potential improvement of our method. The conclusions and future work are given in Section 6.

## 2. Related Work

Within the field of object recognition, some multi-scale CNNs have been proposed. A single classifier is built and rescale the image multiple times to meet all possible object sizes [26]. Representation from multiple stages in the classifier were combined to provide different scales of receptive fields [27]. Feature maps at intermediate network layers were exploited to cover a large range of object sizes [28]. The discussed above CNNs perform multi-scale learning outside the neural network, which means the above CNNs learn features from multi-scale input images or combine the output of intermediate layers. Liao proposed competitive Multi-scale CNN for image classification [29]. 

For image super-resolution, image details are too precious to afford any losses caused by resizing, thus the image details should be extracted by performing multi-scale convolutional filter inside the network.

SRCNN is one of the successful method for image super-resolution with a convolutional neural network. The network builds an end-to-end mapping between a pair of a LR image *Y* and a HR image *X*. Given any size image *Y*, SRCNN can directly output the HR image F(Y).

SCRNN consists of three convolutional layers, and each layer performs one specific task. The *l*-th convolutional layer convolves the image with a set of filters that have the same size $f_{l}\times f_{l}$.

(1) $$Y_{l}=F_{l}\left(Y_{l-1}\right)=max\left(0,W_{l}*Y_{l-1}+B_{l}\right),\;l\in \left\{1,2,3\right\}$$

where $W_{l}$ and $b_{l}$ denote the convolutional filters and biases of the *l*-th layer, respectively, and ’*’ represents the convolutional. $Y_{l-1}$ indicates the input data from the previous layer, and $Y_{l}$ is the output of the convolution. $Y_{0}$ is the original LR images. More detailed structures of CNN in superresolution are summarized below.

- $W_{1}$ corresponds to $n_{1}$ filters of a size of $c\times f_{1}\times f_{1}$, where *c* is the number of image channels and $f_{1}$ is the spatial size of the filter. The output of the first convolution layer is $n_{1}$ feature maps to extract and represent each patch as a high-dimensional feature vector.
- The second convolutional layer is responsible for non-linear mapping. Suppose that we obtain $n_{1}$ dimensional vectors at the above step, the second layer applies $n_{2}$ filters of size $n_{1}\times f_{2}\times f_{2}$ on each feature map. The output $n_{2}$-dimensional vectors will be used for reconstruction.
- The last layer is expected to reconstruct the final HR image by recombining the above high-dimensional patch-wise representations.

Motivated by different tasks, the above three operations all lead to the same form as a convolutional layer. The layers following the first two convolutional layers are rectified linear layers which use a rectified linear unit as an activation function to decide which neuron is fired. Specifically, the 9-5-5 network refers to network $f_{1}=9\times 9$, $f_{2}=5\times 5$, and $f_{3}=5\times 5$ in each convolutional layer. The sizes of the image contexts for reconstruction are decided by the receptive field of CNN. One pixel of F(Y) is reconstructed by 17×17 pixels within the neighborhood from *Y*.

**Figure 1:**
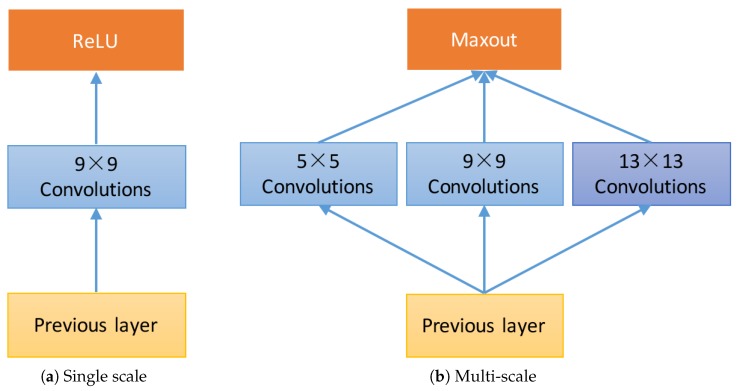
The single scale convolutional layer and the proposed multi-scale competitive module are depicted in (**a**) and (**b**), where (**a**) only contains single scale convolutional filters within each module, whereas (**b**) contains three scale of convolutional kernel.

We introduce a new module that is composed of multi-scale convolutional filters joined by a competitive activation unit. Figure 1 shows the different network modules between SRCNN and the proposed method.

## 3. Proposed Method

**Figure 2:**
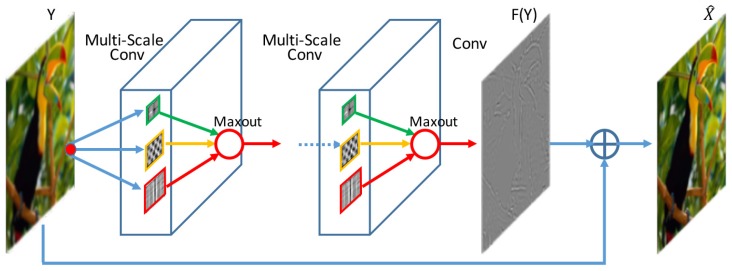
Architecture of the proposed network.

We first introduce the architecture of our network and then describe the implementation details. An overview flowchart of the proposed network is presented in Figure 2.

### 3.1. Multi-scale Competitive Module

Assume that the output of previous layer is $Y_{l-1}$, which consists of $n_{l-1}$ feature maps ($n_{l-1}$ channels), the multi-scale filters are first applied to the input data to produce a set of feature maps $z_{l_{k}}$.

(2) $$z_{l_{k}}=W_{l_{k}}*\left(Y_{l-1}\right)+B_{l_{k}}$$

where $W_{l_{k}}$ corresponds to the *k*-th type filter that contains $n_{l}$ filters of size $n_{l-1}\times f_{l_{k}}\times f_{l_{k}}$. Each convolution produces $n_{l}$ feature maps. Thus, the result of multi-scale convolution consists of $K\times n_{l}$ feature maps.

Second, all the feature maps are divided into non-overlapping $n_{l}$ groups, thus the *i*-th group consists of *K* feature maps $z_{l_{1}}^{i},z_{l_{2}}^{i},\ldots ,z_{l_{K}}^{i}$, and then the maxout function [30] performs maximum pool across $z_{l_{1}}^{i},z_{l_{2}}^{i},\ldots ,z_{l_{K}}^{i}$. The output of the maxout of *i*-th group at position (x,y) is expressed as

(3) $$Y_{l}^{i}\left(x,y\right)=\sigma \left(z_{l_{1}}^{i}\left(x,y\right),z_{l_{2}}^{i}\left(x,y\right),\ldots ,z_{l_{K}}^{i}\left(x,y\right)\right)$$

where σ(·) represents the maxout activation function. For the *i*-th group, that $z_{l_{j}}^{i}\left(x,y\right)$ refers to data at a particular position (x,y) in the *j*-th feature map.

**Figure 3:**
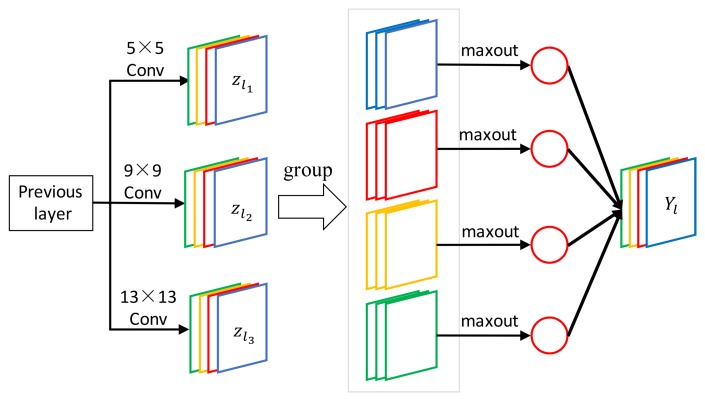
The proposed module which produces the maxout results of multi-scale convolutional.

As shown in Figure 3, the multi-scale convolutional layer includes K=3 types of filter $f_{l_{1}}=5\times 5,\;f_{l_{2}}=9\times 9$ and $f_{l_{3}}=13\times 13$. Suppose that each filter bank contains $n_{l_{1}}=n_{l_{2}}=n_{l_{3}}=4$, and the convolutional output is 12 feature maps divided into 4 groups, the maxout function performs maximum element-wise pooling across these 4 groups of feature maps. In each iteration during the training procedure, the convolutional layer feeds feature maps into the maxout activation function, whereas the activation function ensures that the units that have the maximum values in the group are activated. The final output is 4 feature maps. Specifically, we denote the multi-scale convolutional layer as {5,9,13} when $f_{l_{1}}=13,\;f_{l_{2}}=9$ and $f_{l_{3}}=5$.

Our model is inspired by the structure of SRCNN but differs as follows:

- Multi-scale filters are applied on the input image, which produce a set of feature maps to provide different range of image context for image super-resolution. On the contrary, SRCNN only implements single scale receptive field and provides fixed range of contextual information.
- Competitive strategy is introduced to the activation function. The activation function of SRCNN is ReLU, which is replaced by maxout in our network. The maxout unit reduces the dimensionality of the joint filter outputs and promotes competition among the multi-scale filters.
- A shortcut connection with identity mapping is used to add the input image to the output of the last layer. The shortcut connections can effectively facilitate gradient flow through multiple layers. Thus accelerating deep network training [31].

Compared with competitive multi-scale CNN for image classification in [29], we design the module for image super-resolution. By removing the Batch Normalization (BN) layers, our method not only makes the network suit for image reconstruction, but also saves more GPU memory to build up a deeper model under limited computational resources. The experimental results show that the improvement of performance without BN layer as detailed in Section 5.3.

### 3.2. Training and Prediction

The training procession is to learn the end-to-end mapping function *F* from training samples.

#### 3.2.1. The Loss Function

We now describe the object function of our model. Suppose that *Y* is the input low-resolution image and *X* is the ground-truth high-resolution image; Θ are the network parameters and F(Y;Θ) is the network prediction. We adopt residual learning [19,31] and reformulate the layers as learning residual functions with reference to the layer inputs rather than learning unreferenced functions. Let $\hat{X}$ denote the final output of network, the loss function of the residual estimation is defined as

(4) $$\frac{1}{2}∥X-\hat{X}{∥}^{2}=\frac{1}{2}{∥X-(Y+F(Y;\Theta ))∥}^{2}=\frac{1}{2}{∥R-F(Y;\Theta )∥}^{2}$$

The loss refers to the Euclidean distance between the reconstructed image (the sum of *Y* and F(Y)) and ground truth. Given *N* pairs of LR $\left\{Y_{i}\right\}$ and HR $\left\{X_{i}\right\}$, the loss function is average across all pairs :

(5) $$L\left(\Theta \right)=\frac{1}{N}\sum_{i=1}^{N}∥R_{i}-F_{i}\left(Y_{i};\Theta \right){∥}^{2}$$

where *N* is the number of training samples.

#### 3.2.2. Training

The loss is minimized using stochastic gradient descent with standard back-propagation [32]. In particular, the $W_{l}$ of the convolutional layers are updated as

(6) $$\Delta_{i+1}=\gamma \cdot \Delta_{i}-\eta \cdot \frac{\partial L}{\partial W_{i}^{l}}$$

(7) $$W_{i+1}^{l}=W_{i}^{l}+\Delta_{i+1}$$

where γ is momentum; *l* and *i* are the indices of layers and iterations, respectively; η is the learning rate; and $\frac{\partial L}{\partial W_{i}^{l}}$ is the derivative.

Similar to SRCNN, all the convolutional filters are randomly initialized by a standard normal distribution with deviation 0.01 and biases are set to 0. The learning rate of the multi-scale competitive module and the second convolutional layer is $10^{-2}$, and that of the last layer is $10^{-3}$. The batch size is 32 and the momentum γ is 0.9.

#### 3.2.3. Prediction

In the prediction phase, LR image *Y* is fed into the network, and the prediction result of the network is F(Y). Therefore, the HR image is the sum of the network input *Y* and output F(Y), namely, F(Y)+Y.

### 3.3. Model Properties

#### 3.3.1. Multi-Scale Receptive Fields

For 9-5-5 SRCNN, one pixel of F(Y) is reconstructed by 17×17 pixels within the neighborhood from *Y*. The proposed network can be unfolded to a group of subnetworks that are joined by a maxout unit. For example, {5,9,13}-5-5 can be unfolded into 5-5-5, 9-5-5 and 13-5-5 subnetworks, which implement three sizes of receptive fields: 13×13, 17×17 and 21×21. Furthermore, there is a shortcut connection which skips intermediate layers and adds *Y* to F(Y) directly. This shortcut connection indicates a 1×1 receptive field. Consequently, the proposed network implicitly encodes short- and long-range context information in HR reconstruction. In contrast to a single-scale receptive field of SRCNN, the proposed network provides multi-scale context and improves the flexibility of the network.

#### 3.3.2. Competitive Unit Prevents Filter Co-adaptation

Co-adaptation is a sign of overfitting. Neural units are expected to independently extract features from their inputs rather than relying on other neurons to do so [33]. Imposing the maxout competitive unit to different scale filters explicitly drops the border connections, which not only reduces the chances that these filters will converge to similar regions of the feature space but also protects the 2D structure of the convolutional filters [29].

#### 3.3.3. Fewer Parameters

Suppose that the network consists of a sequence of convolutional layers without pooling and a full connection layer, the number of parameters of convolutional kernels is then computed

(8) $$params_{num}=\sum_{l=1}^{N}\left(f_{l}\times f_{l}\times n_{l}\times n_{l-1}\right);\;n_{0}=1$$

Table 1 shows the number of parameters in four types of networks: 9-5-5, 13-7-5, {5,9,13}-5-5, and {5,9,13}-7-5. In SRCNN, 9-5-5 and 13-7-5, $n_{1}=192$, $n_{2}=64$ and $n_{3}=1$. In multi-scale CNN, $n_{1_{1}}=64,n_{1_{2}}=64,n_{1_{3}}=64$, $n_{2}=64$ and $n_{3}=1$. The 13-7-5 indicates the largest network because all 192-dimensional feature maps are delivered to the second layer. Thanks to the maxout unit, the proposed network reduces the dimensionality of the outputs of the multi-scale convolution, and the parameters of {5,9,13}-5-5 and {5,9,13}-7-5 are much smaller than the others but not sacrifices the super-resolution performance. Although with smaller parameters, our network achieves competitive performance with SRCNN. We present the results of the comparison in the next section.

## 4. Experimental Section

In this section, we first describe how to construct the training datasets. Next, we explore the different structures of the network and then investigate the relation between performance and parameters. Last, we compare our model with SRCNN and other state-of-the-art methods.

### 4.1. Datasets and Evaluation Criteria

**Figure 4:**
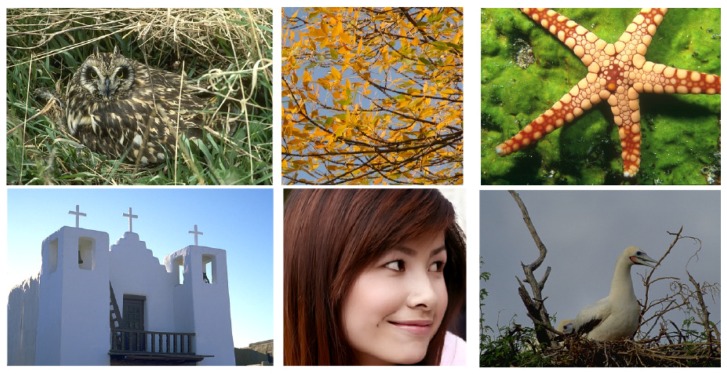
Samples of training images.

The training set consists of 91 images from [6] with the addition of 200 images from the Berkeley Segmentation Dataset [34]. The size of the training samples is 33 for upscale factor 3 and 32 for upscale factor 2 and 4. We extract samples from the original images with a stride of 10, and then we randomly choose 300,000 samples as training samples. Figure 4 shows some training samples. Samples treated as the ground-truth images *X*. To synthesize the LR samples, these samples are first downsampled by a given upscaling factor, and then these LR samples are upscaled by the same factor via Bicubic interpolation to form the LR images. Following [10], super-resolution is only applied on the luminance channel (Y channel in YCbCr color space). Note that all the convolutional layers are padded with zeros before performing convolution to obtain the same size output.

To evaluate our approach, we adopt the peak signal-to-noise ratio (PSNR) and the structural similarity (SSIM) index as evaluation criteria. The benchmark database includes Set5 and Set14 from [9]. The two datasets consist of different types of images; some images include repetitive patterns, such as the face of “Baby” and the tablecloth of “Barbara”, whereas some contain rich structural information, such as “Bird” and “Butterfly”.

We reported the computation time of training and prediction in Table 2, showing that our method increased the training time by a factor of 1.42 and the prediction time by a factor of 1.87. Table 3 summarizes the memory cost. The proposed method requires 266% of the memory consumed by SRCNN, no matter at the phases of training or prediction. Both the time cost and the memory cost of the proposed method are acceptable for image super-resolution.

### 4.2. Parameters and Performance

For fair comparisons, we applied residual learning to the original SRCNN. In the following, all the SRCNN means the residual learning SRCNN. The same training sets, learning rates and initial parameters are used in SRCNN and our proposed model, and all networks are evaluated on Set5 with an upscaling factor of 4.

#### 4.2.1. Filter Size and Performance

**Figure 5:**
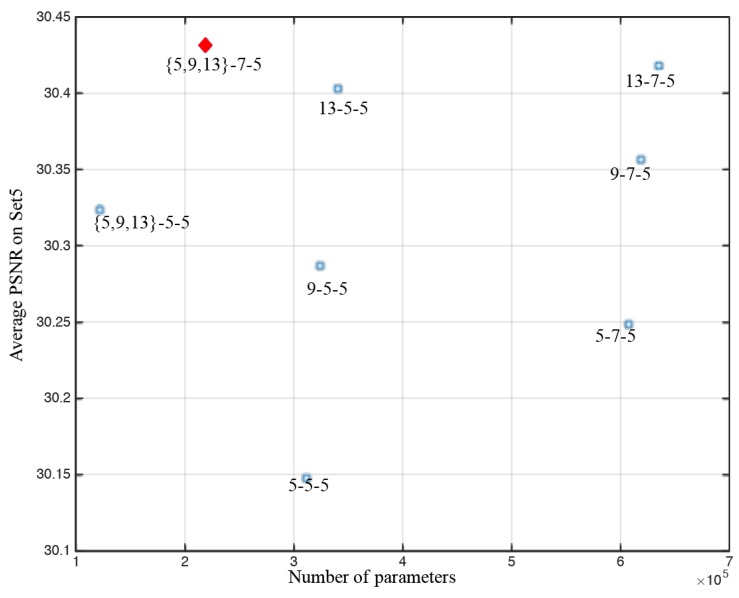
Different filter size and performance on Set5 with upscaling factor 4. The proposed method achieve trade-offs between performance and parameter sizes.

Based on the basic 5-5-5 network, we progressively modify the filter size to investigate the relations between performance and filter sizes. Figure 5 shows the average PSNR (Set5) of the networks trained after 80 epochs. The filter numbers of SRCNN are $n_{1}=192,$ $n_{2}=64$, and $n_{3}=1$. {5,9,13}-5-5, which includes three scales and performs better than the single-scale networks: 5-5-5, 9-5-5 and 13-5-5; {5,9,13}-7-5 performs better than 5-7-5, 9-7-5 and 13-7-5.

A reasonable larger filter size can improve reconstruction performance. As shown in Table 4, when the filter size is increased from {3,5,9} to {5,9,13} or even larger size, the PSNR has been increased from 30.10 dB to 30.44 dB. Too large size, e.g., {7,13,15}, will degrade the performance and introduce more computation. Therefore, moderate filter size such as {5,9,13} is suggested.

#### 4.2.2. Epoch and Performance

**Figure 6:**
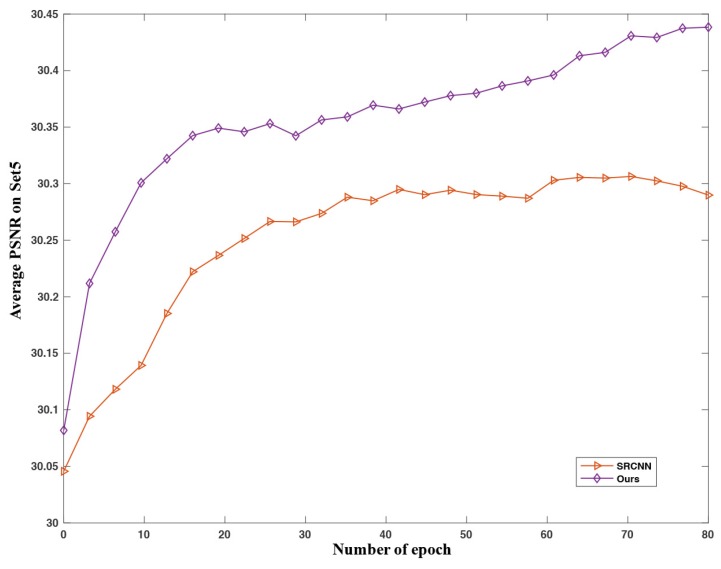
Convergence curve of different networks on Set5 with upscaling factor 4. The proposed networks achieve better performance than SRCNN after a few epochs.

As illustrated in Figure 6, the proposed networks quickly reach state-of-the-art performance within a few epochs. Our model {5,9,13}-7-5 progressively improve over time. We will show that better results can be obtained by providing a longer training time in the following experiments.

### 4.3. Results

We compare the proposed method with state-of-the-art methods both qualitatively and quantitatively. The compared methods include the baseline method Bicubic, adjusted anchored neighborhood regression method (A+) [10], and three CNN-based methods: SRCNN [17], CSCN [36] and FSRCNN [37]. For fair comparisons with SRCNN which training data include 5 million sub-images from imageNet, we augment the training set to 1 million sub-images by rotating and flipping the 300,000 images in the original training set. Table 5 illustrates the average quantitative performance of the compared methods. The proposed method outperforms the other methods for most of images. Furthermore, the PSNR has been increased about 0.1 to 0.4 dB when we build up a deeper network by stacking two modules.

**Figure 7:**
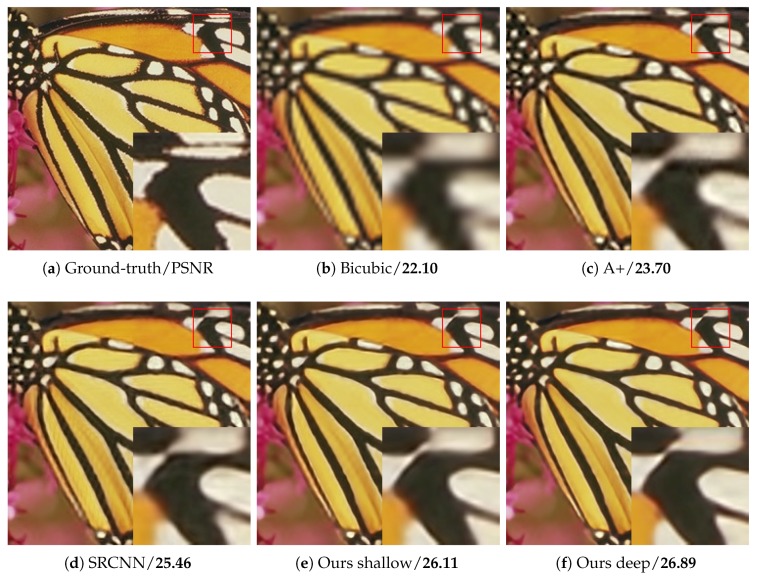
The “Butterfly” images with an upscaling factor 4.

**Figure 8:**
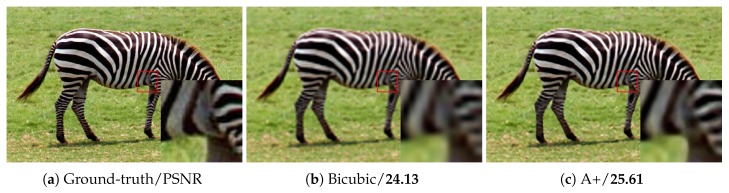

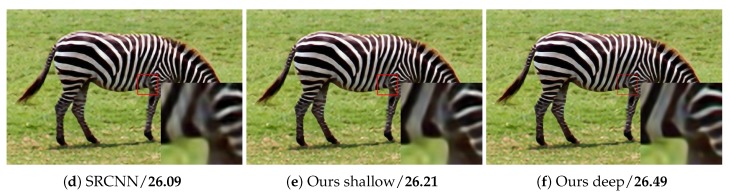
The “Zebra” images with an upscaling factor 4.

**Figure 9:**
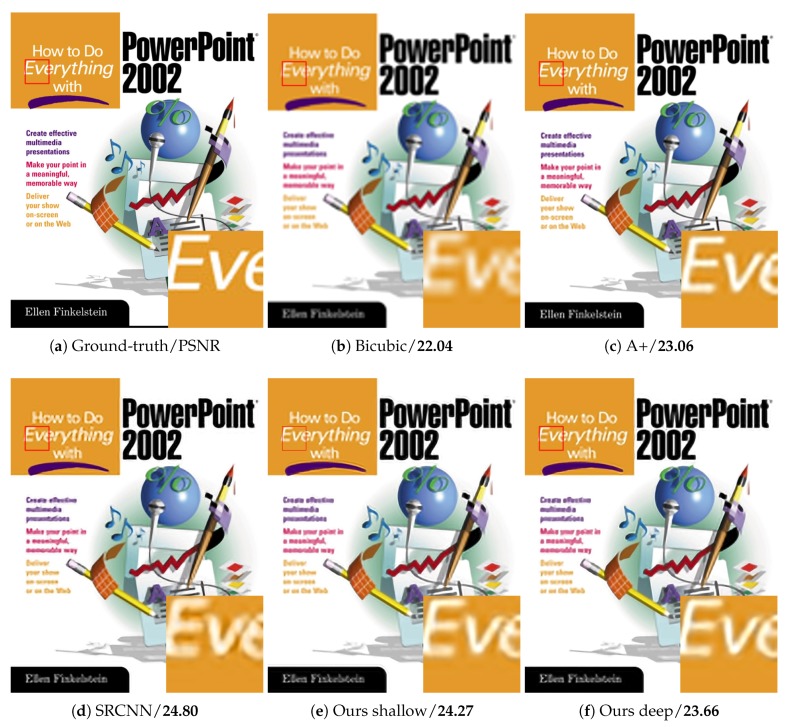
The “PPT3” images with an upscaling factor 4.

**Figure 10:**
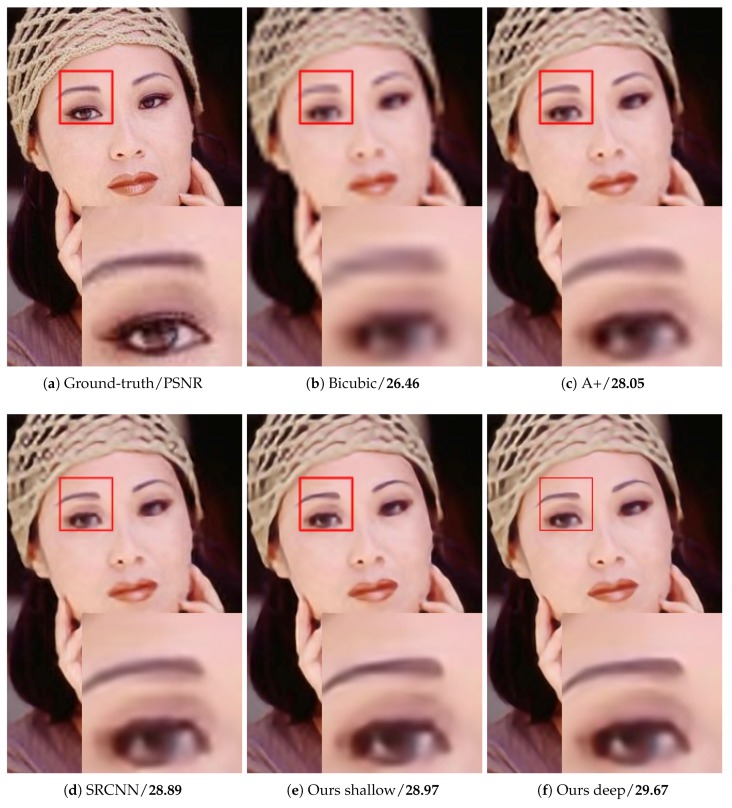
The “Woman” image with an upscaling factor 4.

As shown in Table 6, our method surpasses SRCNN largely on “Butterfly”, “Woman”, “Bird” and “Monarch”, which have rich image details and diverse image features. For images with a large smooth region or repetitive texture pattern, such as “Baby”, “PPT3” and “Barbara”, the PSNR of our method is lower than that of the other methods. By stacking more multi-scale competitive module, the network provides more various size context for image reconstruction. Table 6 illustrates the better performance of our deep network than our shallow one. Figure 7, Figure 8, Figure 9 and Figure 10 present some sampled results generated by the compared methods. The HR images restored by the proposed method are perceptually more plausible with relatively sharp edges and few artifacts.

## 5. Discussions

In this section, we compared our method to other state-of-art methods on large datasets. Furthermore, we also show the potential improvement of performance by Iterative Back-Projection (IBP) filter [4].

### 5.1. Comparison with Other State-of-Art Methods

We choose to compare against the best SRCNN (9-5-5), SRGAN [38], ESPCN [39] and VDSR [19] on larger datasets: BSD300 and BSD500 [34]. The results are shown in Table 7.

We divide these compared methods into two types, shallow network that contains only 3-4 layers and much deeper network that have more than 16 layers. The former type includes SRCNN, ESPCN and the proposed methods while the latter one includes SRGAN and VDSR. Our method achieves the best evaluation criteria among all the shallow networks and also obtains better performance than a deeper network, SRGAN, which has 16 layers. A much deeper network, VDSR, obtains the best performance among all compared methods but its network has 20 layers. Overall, our method is a better choice under limited computational resources.

### 5.2. Improvement with Iterative Back Projection

The iterative back projection (IBP) refinement generally improves the PSNR as it makes the HR reconstruction consistent with the LR input and the employed degradation operators. We perform IBP as post-process of our method, and Table 8 shows the improvements obtained with iterative back projection refinement.

### 5.3. The Effect of Batch Normalization on Super-Resolution

Reference [29] proposed competitive network with Batch Normalization for image classification. Reference [29] solved the problem of image classification while our work is designed for image super-resolution. Therefore, we analyzed the effect of BN in image super-resolution.

We removed the Batch Normalization (BN) layers from our module and attained better performance in terms of higher PSNR and SSIM criteria, as shown in Table 9. Experiments proved that even with deeper network, BN still reduces the super-resolution performance [42]. In addition, the BN layers consume more GPU memory to restore the results of BN layers. Thus, our method is more convenient to build up a deeper model under limited computational resources.

## 6. Conclusions

We propose a super-resolution reconstruction model for single images based on multi-scale convolutional neural network. Moreover, large filters and small filters are jointly trained within the same model. The maxout unit not only reduces the dimensionality of the filter outputs, but also promotes competition among the multi-scale filters. The success of the proposed network is due to its ability to provide a multi-range of context and adaptively select the optimal local receptive field for image reconstruction. Experiments on super-resolution illustrate the high performance of our network. Under limited computational resources, our method achieves the best evaluation criteria among all the shallow networks and also obtains better performance than a deeper network. The experiments demonstrate that our method can fully take advantage of the cost/accuracy trade-off. The further improvement is expected when stacking more multi-scale competitive modules.

## Figures and Tables

**Table 1:** Number of parameters in different networks.

Network	Parameter Number
9-5-5	324,352
13-7-5	636,160
{5,9,13}-5-5	123,600
{5,9,13}-7-5	219,904

**Table 2:** The computation time of training phase and prediction phase.

Time	SRCNN (9-5-5)	Ours ({5,9,13}-7-5)
Training with GPU (one epoch)	12 min	15 min
Prediction with CPU (256×256)	0.30 s	0.56 s

Note: Both methods have 3 layers. Our model is implemented using the MatConvNet package [35] on a work-station with Intel 3.4 GHz CPU, GTX960 GPU and 16GB memory. The training data consists of 300,000 samples and batch size is 32. Due to limited memory of GTX960, the prediction runs under CPU mode.

**Table 3:** Memory cost for different networks.

Network	SRCNN (9-5-5)	Ours ({5,9,13}-7-5)
Amount of parameters	224 KB	465 KB
Memory cost (training)	24 MB	64 MB
Memory cost (prediction)	49 MB	129 MB

Note: In training phase, the batch size is 32 and the sample size is 32×32. The prediction runs under CPU mode, the input image size is 256×256. Both parameters and image are stored in float32. The parameter numbers and memory costs are calculated by vl_simplenn_display function of MatConvNet package.

**Table 4:** PSNR performance under different filter size on Set5 with upscaling factor 4.

Proposed Method	PSNR
{3,5,9}-7-5	30.10
{5,9,13}-7-5	30.44
{7,9,13}-7-5	30.44
{5,11,13}-7-5	30.43
{7,9,15}-7-5	30.33
{7,13,15}-7-5	30.27

**Table 5:** The results of PSNR (dB) and SSIM on two test datasets. Red indicates the best and blue indicates the second best performance.

Dataset Upscaling Factor	Set5 2	Set14 2	Set5 3	Set14 3	Set5 4	Set14 4
**Bicubic**	33.66/0.9299	30.33/0.8694	30.39/0.8681	27.61/0.7752	28.42/0.8104	26.06/0.7042
**A+**	35.95/0.9508	31.84/0.9017	32.07/0.8984	28.71/0.8121	29.83/0.8465	27.02/0.7421
**SRCNN**	36.66/0.9542	32.55/0.9073	32.75/0.9090	29.39/0.8225	30.49/0.8628	27.60/0.7529
**CSCN**	36.93/0.9552	32.56/0.9074	33.10/0.9144	29.41/0.8238	30.86/0.8732	27.64/0.7578
**FSRCNN**	37.00/0.9558	32.63/0.9088	33.16/0.9140	29.43/0.8242	30.71/0.8657	27.59/0.7535
**Ours (shallow)**	37.03/0.9566	32.69/0.9103	33.04/0.9140	29.43/0.8258	30.72/0.8706	27.68/0.7581
**Ours (deep)**	37.23/0.9575	32.73/0.9222	33.44/0.9185	29.58/0.8290	31.10/0.8785	27.79/0.7627

Note: We build two networks for image super-resolution, the shallow one is {5,9,13}-7-5 and the deep one refers to {5,7,9}-{5,7,9}-5-5 with two multi-scale competitive modules and two single scale convolutional layers.

**Table 6:** The detail results of PSNR (dB) and SSIM on two test datasets for upscale 4x. Red indicates the best and blue indicates the second best performance.

	Bicubic	A+	SRCNN	Ours (Shallow)	Ours (Deep)
Image	PSNR/SSIM	PSNR/SSIM	PSNR/SSIM	PSNR/SSIM	PSNR/SSIM
baby	31.78/0.8567	33.07/0.8811	33.13/0.8824	32.75/0.8807	33.06/0.8824
bird	30.18/0.8729	32.03/0.9048	32.52/0.9112	32.76/0.9163	33.22/0.9232
butterfly	22.10/0.7369	23.70/0.8023	25.46/0.8566	26.11/0.8801	26.89/0.8987
head	31.59/0.7536	32.30/0.7771	32.44/0.7801	32.49/0.7836	32.67/0.7884
woman	26.46/0.8318	28.05/0.8670	28.89/0.8837	28.97/0.8893	29.67/0.8996
baboon	22.41/0.4521	22.71/0.5002	22.73/0.5029	22.76/0.5112	22.80/0.5128
barbara	25.17/0.6873	25.68/0.7245	25.76/0.7293	25.74/0.7346	25.94/0.7413
bridge	24.44/0.5652	24.01/0.6233	25.11/0.6220	25.17/0.6288	25.28/0.6332
coastguard	25.38/0.5238	25.80/0.5539	26.04/0.5563	26.09/0.5623	26.16/0.5667
comic	21.72/0.5852	22.41/0.6454	22.70/0.6658	22.76/0.6773	22.88/0.6864
face	31.60/0.753	32.27/0.7757	32.38/0.7779	32.42/0.7808	32.61/0.7862
flowers	25.59/0.7233	26.62/0.7648	27.14/0.7791	27.21/0.7856	27.20/0.7901
foreman	28.79/0.8625	31.22/0.8927	32.14/0.9080	32.14/0.9109	32.30/0.9146
lenna	29.87/0.8149	31.18/0.8416	31.41/0.8436	31.51/0.8481	31.79/0.8522
man	25.72/0.6760	26.52/0.7182	26.89/0.7300	27.00/0.7370	27.14/0.7428
monarch	27.51/0.8817	28.88/0.9037	30.22/0.9181	30.76/0.9251	31.35/0.9312
pepper	30.42/0.8359	32.28/0.8583	32.98/0.8648	32.95/0.8674	33.48/0.8720
ppt3	22.04/0.8151	23.06/0.8473	24.80/0.8928	24.27/0.8871	23.66/0.8867
zebra	24.13/0.6831	25.61/0.7391	26.09/0.7505	26.21/0.7605	26.49/0.7615

**Table 7:** The mean PSNR (dB) obtained with different methods.

Dataset	Upscaling	Shallow Network			Much Deeper Network	
	Factor	Proposed Method	SRCNN	ESPCN	SRGAN	VDSR
BSD300	2	**31.62**	31.31	N/A	N/A	**31.90**
	3	**28.60**	28.37	28.54	N/A	**28.82**
	4	**27.07**	26.87	27.06	25.16	**27.29**
BSD500	2	**31.95**	31.58	N/A	N/A	**32.27**
	3	**28.72**	28.45	28.64	N/A	**28.95**
	4	**27.10**	26.90	27.07	N/A	**27.31**
Set5	2	**37.23**	36.66	N/A	N/A	**37.53**
	3	**33.44**	32.75	33.13	N/A	**33.66**
	4	**31.10**	30.49	30.90	29.40	**31.35**
Set14	2	**32.73**	32.55	N/A	N/A	**33.03**
	3	**29.58**	29.39	29.49	N/A	**29.77**
	4	**27.79**	27.60	27.73	26.02	**28.01**

Note: For SRCNN and VDSR, we use its trained model and source codes [40,41] to do the super-resolution on our hardware. The criteria of SRGAN and ESPCN are cited from [38,39] respectively. Best criteria in each category are shown in bold. N/A indicates the results not provided by author.

**Table 8:** Performance improvement with iterative back projection (IBP).

Upscaling		Proposed Method	
Factor	Dateset	Without IBP	With IBP
2	Set5	37.23/0.9575	**37.28/0.9579**
	Set14	32.73/0.9222	**32.89/0.9110**
3	Set5	33.44/0.9185	**33.53/0.9195**
	Set14	29.58/0.8290	**29.70/0.8296**
4	Set5	31.10/0.8785	**31.19/0.8800**
	Set14	27.79/0.7627	**27.86/0.7632**

Note: The number of iterations in IBP is 5. Better criteria are marked in bold.

**Table 9:** The effect of BN of our method.

Upscaling		Proposed Method	
Factor	Dateset	Without BN	With BN
2	Set5	**37.03/0.9566**	36.90/0.9556
	Set14	**32.69/0.9103**	32.62/0.9087
3	Set5	**33.04/0.9140**	32.88/0.9117
	Set14	**29.43/0.8258**	29.35/0.8236
4	Set5	**30.72/0.8706**	30.56/0.8663
	Set14	**27.68/0.7581**	27.59/0.7542

Note: Our module is {5,9,13}-7-5 and the network with BN is {5-BN, 9-BN, 13-BN}-7-5. Better criteria are marked in bold.
